# Gold, silver and nickel nanoparticle anchored cellulose nanofiber composites as highly active catalysts for the rapid and selective reduction of nitrophenols in water†

**DOI:** 10.1039/c7ra10489h

**Published:** 2018-01-17

**Authors:** Mayakrishnan Gopiraman, Dian Deng, Somasundaram Saravanamoorthy, Ill-Min Chung, Ick Soo Kim

**Affiliations:** Department of Applied Bioscience, College of Life & Environment Science, Konkuk University 120 Neungdong-ro, Gwangjin-gu Seoul 05029 South Korea imcim@konkuk.ac.kr illminchung@gmail.com; Nano Fusion Technology Research Group, Division of Frontier Fibers, Institute for Fiber Engineering (IFES), Interdisciplinary Cluster for Cutting Edge Research (ICCER), Shinshu University Tokida 3-15-1 Ueda Nagano prefecture 386-8567 Japan kimicksoo.gr@gmail.com; Department of Chemistry, National Institute of Technology Tiruchirappalli 620 015 India

## Abstract

Highly active metal nanoparticle (MNP) supported cellulose nanofiber (CNF) composites (Au/CNF, Ni/CNF and Ag/CNF) were prepared for the reduction of 4- and 2-nitrophenols (4-NP and 2-NP) in water. Transmission electron microscopy (TEM) images showed that the ultrafine nanoparticles (Au, Ni and Ag NPs) were uniformly deposited on CNFs surface. The content of Au (9.7 wt%), Ni (21.5 wt%) and Ag (22.6 wt%) in Au/CNF, Ni/CNF and Ag/CNF respectively was determined by energy dispersive spectroscopy (EDS) and inductive coupled plasma-mass spectroscopy (ICP-MS) analysis. The chemical state of the MNPs in Au/CNF, Ni/CNF and Ag/CNF was determined by X-ray photoelectron spectroscopy (XPS) and X-ray diffraction (XRD). The significant metal-support interaction was studied by means of XPS. The Au/CNF, Ni/CNF and Ag/CNF demonstrated excellent catalytic activity towards the reduction of nitrophenols to aminophenols in water. To our delight, even a very low amount of catalyst was also found to be good enough to achieve 100% reduction of 4- and 2-NP with a higher reaction rate (within 5 min). The best rate constant (*k*_app_) values were determined for the cellulose nanocomposites. To the best our knowledge, Au/CNF, Ni/CNF and Ag/CNF are the most efficient nanocatalysts for the reduction of 4- and 2-NP reported to date. The catalytic performance of Au/CNF, Ni/CNF and Ag/CNF was compared with previously reported results. A possible mechanism has been proposed for these catalytic systems.

## Abstract 2

Metal nanoparticles supported cellulose nanofiber composites (Au/CNF, Ni/CNF and Ag/CNF) were found to be highly efficient nanocatalysts for the rapid and selective reduction of nitrophenols in water.

## Graphical abstract

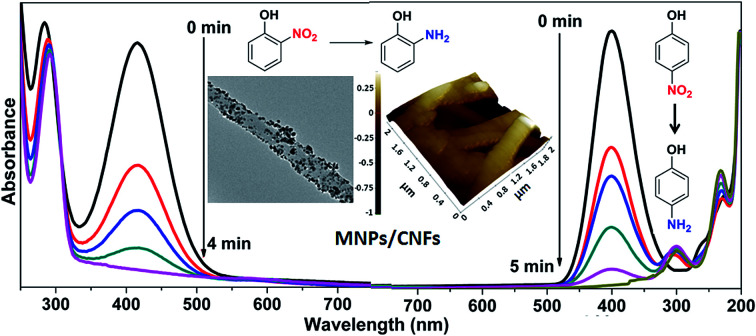

## Introduction

Cellulose nanocomposites have received substantial consideration both in industry and academia due to their enhanced physicochemical properties, unique structural features and wide range of applications.^1^ Particularly, the cellulose nanocomposites produced *via* the electrospinning (ES) method have gained much attention due to their huge surface area, easy handling and exceptional mechanical properties.^2,3^ The role of cellulose nanocomposites in applications such as catalysis, sensors, filters, energy, separators and biomedicine is significant.^4^ Applications of cellulose nanofiber as a metal NP support have attracted a lot of interest in recent years.^5^ Metal NP supported cellulose nanofibers have been used for various applications such as in water purification and separation, sensors, antibacterial treatment, biological and environmental analysis, and catalysis. For instance, Son *et al.*^6^ prepared a cellulose acetate nanofiber/Ag NP nanocomposite and it was used for its antimicrobial activity. Zhang and co-workers^7^ reported biotemplated synthesis of AuNPs/cellulose nanofiber composites. They found that the prepared nanofiber composites showed excellent results in biosensing applications. Similarly, Ag nanoparticle-immobilized cellulose nanofibril films were prepared and it was studied for environmental conservation by Ramaraju *et al.*^8^ Very recently, our group has prepared Ag and Ru NPs immobilized cellulose nanofiber composites. We found that the nanocomposites have superior catalytic activity towards the aerobic oxidation of alcohol and *aza*-Michael reaction.^9^ In spite of the advantages, a very limited number of works published in the literature on the cellulose nanofiber composites for catalytic applications. Hence, the present study is focused on the cellulose nanocomposites for catalytic applications.

Nitrophenols including 4-nitrophenol (4-NP) and 2-nitrophenol (2-NP) are a common organic pollutant that occurs in industrial wastewater.^10^ Generally, the nitrophenols are very stable in wastewater and often cause environmental pollution due to its carcinogenesis, hepatotoxicity and mutagenesis.^11^ Hence, the removal of nitrophenols from wastewater is always an important issue. Traditionally, the nitrophenols were removed from wastewater by several methods including electrocoagulation, adsorption, photocatalytic degradation, microbial degradation, electrochemical treatment, electro-Fenton method, electrocoagulation, and so on.^12^ However, recently, the conversion of waste nitrophenols into valuable compounds, aminophenols, has gained vast attention.^13^ The aminophenols are very useful in various applications such as photographic developer, corrosion inhibitor, anticorrosion-lubricant, analgesic and antipyretic drugs.^14^ Commonly, the metal NPs are dispersed onto solid matrices to achieve better performance and easy recovery. Common solid matrices such as carbon, silica, titania, alumina, magnesia, and zirconia and *etc.*, are often used to prepare the heterogeneous metal NPs catalysts.^15^ Due to unique and facile surface modification, the cellulose nanofiber can be an efficient solid support for the controlled immobilization of metal NPs.^16,17^ So far, supported Au, Ag and Ni NPs are found to be the most efficient catalyst for the conversion of nitrophenols to aminophenols.^13^ We presume that the Au, Ag and Ni NPs supported cellulose nanocomposite would show an excellent activity towards to the reduction of 2- and 4-NP. Our aim is to use cellulose nanocomposites as catalyst for the conversion of nitrophenols to aminophenols. To our delight, this is the first report on the metal-based cellulose nanofiber composite for the nitrophenol reduction. Herein, we prepared Ag, Au and Ni NPs supported cellulose nanofiber composite (Ag/CNF, Au/CNF and Ni/CNF). The resultant Ag/CNF, Au/CNF and Ni/CNF was used for the reduction of 2- and 4-NP.

## Experimental section

### Materials

Gold(iii) chloride (AuCl_3_, 99.9%), silver(i) nitrate (AgNO_3_, ≥99.0%), nickel(ii) chloride (NiCl_2_, 99.9%), sodium hydroxide (NaOH, 99.9%), sodium chloroacetate (ClCH_2_COONa, ≥98%), cellulose acetate (CA, 39.8% acetyl content, *M*_w_ = 30 kDa), acetone (≥99.9%), *N*,*N*-dimethylformamide (DMF, 99.7%), sodium borohydride (NaBH_4_, ≥98.0%) and nitrophenols (≥99%) were purchased from Wako Pure Chemicals, Japan or Sigma-Aldrich, and used as received.

### Preparation of cellulose nanofiber composites

The anionic cellulose nanofiber (m-CNFs) was prepared according to our previous report.^9^ Initially, 18 wt% of cellulose acetate solution was prepared using acetone/DMF mixture (3 : 2 ratios) and it was electrospun under electric field of 12 kV to obtain cellulose acetate nanofibers (CANFs). The resultant CANFs was deacetylated using 0.05 M of NaOH to produce regenerated cellulose nanofibers (CNFs). Subsequently, soda cellulose (Na–CNFs) was produced from CNFs by dipping it into 1.5 M NaOH solution for 5 min. Then, the Na–CNFs were dipped into a 1.0 M ClCH_2_COONa solution for 6 h to obtain anionic cellulose nanofiber (m-CNFs).

**Fig. 1:**
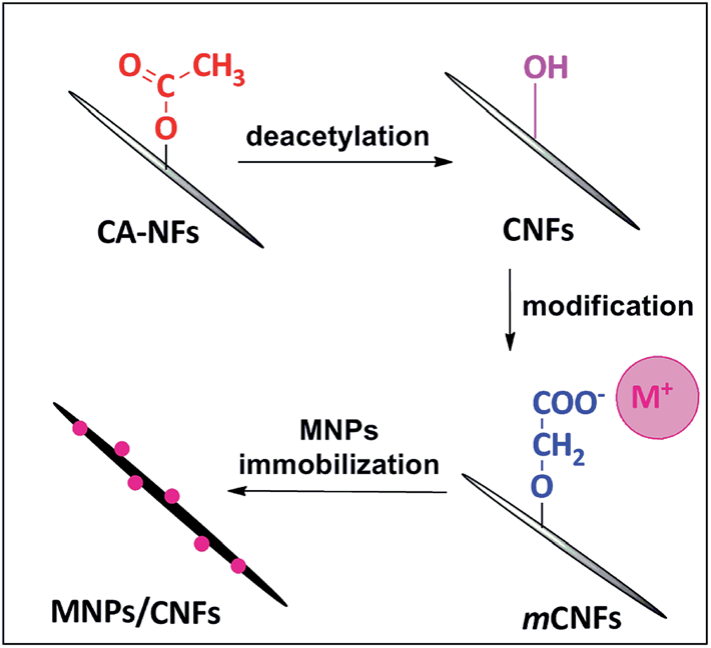
Illustration for the preparation of MNPs/CNFs.

As shown in Fig. 1a, the m-CNFs were used to prepare the cellulose nanofiber composites (Au/CNF, Ni/CNF and Ag/CNF). A simple wet reduction method using NaBH_4_ as a reducing agent was adopted for this purpose. In a typical preparation of Au/CNFs, 100 mg of m-CNF was dipped into a 100 mL of aqueous AuCl_3_ solution at 60 °C for 24 h, followed by gentle washing with distilled water to remove the excess AuCl_3_. The resultant wet mat was treated with NaBH_4_ (1 mM, 20 mL) at 25 °C for 15 min. Similarly, the Ag/CNF and Ni/CNF were prepared by using AgNO_3_ and NiCl_2_ as precursor (100 mL, 0.5 mmol). Finally, the obtained Au/CNF, Ni/CNF and Ag/CNF nanofiber composites were washed with distilled water and air dried.

### Characterization

Electrospinning was carried out by using a high-voltage power supply (Har-100*12, Matsusada Co., Tokyo) as the source of electric field, capable of generating voltages up to 100 kV. Transmission electron microscope (TEM) was performed on JEM-2100 JEOL Japan with accelerating voltage of 120 kV. Scanning electron microscope-energy dispersive spectrum (SEM-EDS, Hitachi, model-3000H) was used to determine the metal content in nanocomposites. Wide angle X-ray diffraction (WAXD) patterns were recorded on Rotaflex RTP300, Rigaku.Co., Japan. Nickel-filtered Cu Kα radiation was used for the measurements, along with an angular range of 10 < 2*θ* < 50°. Fourier transform infrared (FT-IR) spectra were taken out by using IR Prestige-21, Shimadzu, Japan. X-ray photoemission spectra (XPS) were recorded on Kratos Axis-Ultra DLD model instrument. Ultraviolet-visible (UV-vis) spectra were recorded in 3 cm path length quartz cell on a Shimadzu UV-2600 spectrophotometer. The reductive products (2- and 4-aminophenol) were further confirmed by ultra high performance liquid chromatography (Thermo UHPLC electron corporation, accucore C-18 column, solvent system water : methanol 30 : 70 ratio) at 254 nm wavelength.

### Reduction of 4- and 2-nitrophenol

A 40 or 80 μL of aqueous nitrophenol solution (0.01 M) was mixed with 4 mL freshly prepared NaBH_4_ solution (0.01 M). To the above mixture, particular amount of Au/CNFs, Ni/CNFs and Ag/CNFs was added and stirred well. At a given reaction time, the UV-Vis absorption spectra were recorded within the wavelength range of 250–600 nm. The rate constants of the reduction reaction were calculated by measuring the peak intensity evolution every minute at wavelengths of 400 nm and 416 nm for 4-NP and 2-NP, respectively.

## Results and discussion

### Characterization of Au/CNF, Ni/CNF and Ag/CNF composites

In a typical preparation of cellulose nanofibers, cellulose acetate nanofibers (CANFs) were prepared from solution was prepared by electrospinning method. The electrospun CANFs were deacetylated to obtain regenerated cellulose nanofibers (CNFs). Subsequently, the surface of CNFs was chemically modified with anionic group (O–C=O^−^) (Fig. 1). The successful conversion of CANFs to m-CNFs was confirmed by FTIR. The FT-IR spectrum of CANFs (Fig. S1†) showed three intense peaks at 1730 (C=O), 1373 (C–CH_3_) and 1223 cm^−1^ (C–O–C), whereas, after deacetylation, the C=O absorption at 1730 cm^−1^ completely disappeared.^18^ In addition, a new peak at 3400 cm^−1^ (hydroxyl, –OH) was noticed. The FTIR spectrum of m-CNFs showed two new peaks at 1572 (–COO^−^) and 1611 cm^−1^ (C–CH_2_). The result clearly shows the successful surface modification of CNFs. The resultant modified cellulose nanofiber (m-CNFs) was used as a support for the immobilization of metal nanoparticles (Au, Ag and Ni NPs). In fact, the presence of anionic group (O–C=O^−^) would act as an anchoring sites for the metal nanoparticles.^19^ In addition, the anionic groups can form a coordination type of bonding with metal ions, which facilitate the formation of ultrafine nanoparticles with very fine dispersion on the surface of the cellulose nanofibers.

**Fig. 2:**
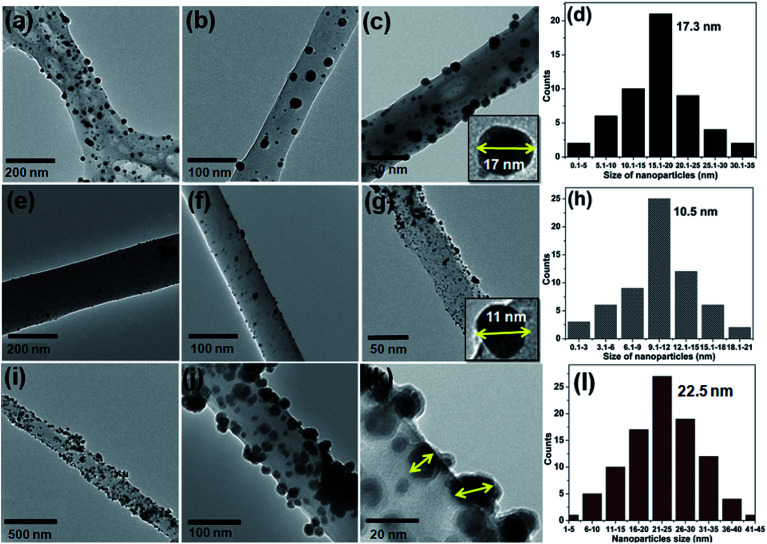
TEM images of ((a–c) Ag/CNF, (e–g) Au/CNF and (i–k) Ni/CNFs); the histogram of nanoparticles diameter distribution for (d) Ag NPs, (h) Au NPs and (l) Ni NPs.

**Fig. 3:**
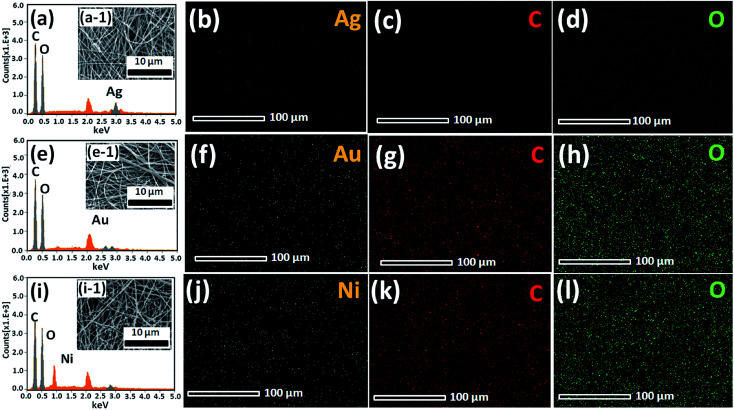
SEM images, EDS spectra, and corresponding elemental mapping of (a, a-1, b, c and d) Ag/CNF, (e, e-1, f, g, h) Au/CNF and (i, i-1, j, k and l) Ni/CNF.

The excellent surface morphology of the nanofiber composites (Au/CNF, Ni/CNF and Ag/CNF) was confirmed by the TEM images (Fig. 2). The results showed that the metal nanoparticles are uniformly dispersed with good adhesion on the CNFs surface. Moreover, the magnified TEM images (Fig. 2c, g and k) of the nanofiber composites confirmed that the metal nanoparticles are attached on the surface of the m-CNFs. In order to calculate the average size of the nanoparticles, fifty individual nanoparticles were chosen and the size was measured. The histogram of nanoparticles diameter distribution for Ag, Au and Ni NPs has been presented in Fig. 2. The average diameter of Ag, Au and Ni NPs was calculated to be 17.3, 10.5 and 22.5 nm respectively. The surface area per unit mass (*S*) of metal nanoparticles was calculated by using the equation *S* = 6000/(*ρ* × *d*), where *ρ* is the density of metal and *d* is the average diameter of metal nanoparticles.^15^ The surface area per unit mass of Au in Au/CNF, Ni in Ni/CNF and Ag in Ag/CNF was calculated to be 29.6, 29.9 and 33.1 m^2^ g^−1^ respectively. The elemental content of the nanofiber composites were determined by SEM-EDS and ICP-MS analysis. Fig. 3 shows the representative SEM, EDS and corresponding elemental mapping of Au/CNF, Ni/CNF and Ag/CNF. In order to determine the factual metal content in the nanocomposites, three different areas where choose to take EDX and the average metal content was calculated (see Table S1 in ESI†). The average content of Au (9.7 wt%), Ni (21.5 wt%) and Ag (22.6 wt%) in Au/CNF, Ni/CNF and Ag/CNF was determined by EDS analysis. Similarly, the ICP-MS results confirmed the metal content of 9.45 (Au), 21.45 (Ni) and 22.94 wt% (Ag) was determined for the Au/CNF, Ni/CNF and Ag/CNF nanocomposites respectively. The elemental mapping reveals the homogeneous dispersion of metal nanoparticles in the nanofiber composites (Fig. 3).

**Fig. 4:**
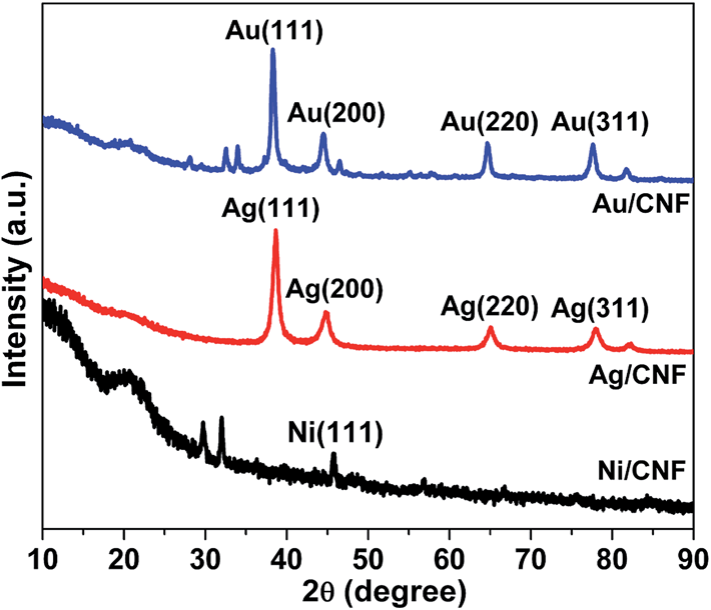
XRD patterns of Ag/CNF, Au/CNF and Ni/CNF.

Chemical state of Ag in Ag/CNF, Au in Au/CNF and Ni in Ni/CNF was confirmed by XRD and XPS analysis. Fig. 4 depicts XRD patterns of Ag/CNF, Au/CNF and Ni/CNF. The XRD pattern of Ag/CNF showed Bragg reflections with 2*θ* values of 38.03°, 46.18°, 63.43° and 77.18° which can be indexed to (1 1 1), (2 0 0), (2 2 0) and (3 1 1) planes of metallic Ag (JCPDS file no. 87-0720).^20^ Alike, four sharp diffraction peaks at around 2*θ* = 38.2°, 2*θ* = 44.4°, 2*θ* = 64.6° and 2*θ* = 77.6° correspond to (111), (200), (220), (311) and (222) facets respectively, indicating the metallic form of Au in Au/CNF (JCPDS file no. 04-0784).^21^ The XRD pattern of Ni/CNF confirmed a sharp diffraction peak at around 2*θ* = 45.8°, which correspond to the (111) reflection of metallic Ni.^22^

**Fig. 5:**
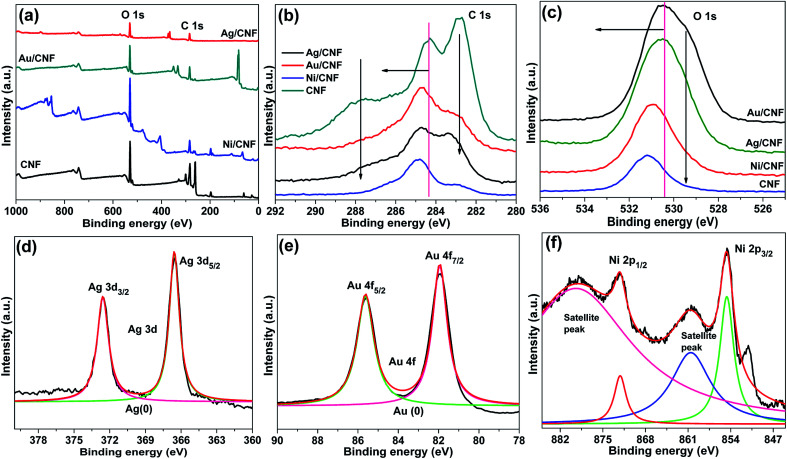
(a) Survey, (b) C 1s and (c) O 1s XPS spectra of Ag/CNF, Au/CNF and Ni/CNF, and (d) Ag 3d peaks of Ag/CNF, (e) Au 4f peaks of Au/CNF and (f) Ni 2p peaks of Ni/CNF.

The XPS spectra were taken for m-CNFs, Ag/CNF, Au/CNF and Ni/CNF; the results are demonstrated in Fig. 5. Two peaks at binding energies (BE) = ∼283.1 eV (C 1s peak) and BE = ∼530.5 eV (O 1s peak) were noticed for all the samples. The C 1s peak at BE = ∼283.1 eV confirms the presence of C–C, C–H, C–OH, C=O and C–O–C groups in the nanofiber composites. Alike, the O 1s spectra showed the existence of C=O and C–OH functional groups.^23^ It is very interesting to note that the C 1s peaks and O 1s peaks of m-CNFs after metal NPs decoration showed a positive shift in the biding energy. The results indicate that the metal NPs were strongly attached on the surface of the cellulose nanofiber. In the case of Ag/CNF, the XPS spectrum showed two new peaks at BE = 367.0 eV (Ag 3d_3/2_) and BE = 373.0 eV (Ag 3d_5/2_). Generally, the metallic Ag appears at BE = 368.1 eV (Ag 3d_3/2_) and BE = 374.1 eV (Ag 3d_5/2_), with Ag 3d doublet slitting of 6.0 eV.^24^ However, in the present case the peaks shifted toward positive side by 1.1 eV compared to metallic Ag, whereas the slitting of 6.0 eV was maintained. The results confirmed the metallic nature of the Ag in Ag/CNF. In case of the 4f region of Au/CNF, a doublet 4f_7/2_ (85.9 eV) and 4f_5/2_ (82.2 eV) was noticed. This doublet was observed to be shifted toward positive side by 1.9 eV compared to Au 4f_7/2_ peak of metallic Au (84.0 eV).^25^ In the Ni 2p_3/2_ spectra of Ni/CNF, the peak at 853.4 ± 0.5 eV corresponding to metallic Ni was noticed. In addition, the satellite peaks at 860.6 and 878.4 eV confirm the presence of small amount of Ni^2+^.^26^

### Catalytic performance of Au/CNF, Ni/CNF and Ag/CNF composites

**Fig. 6:**
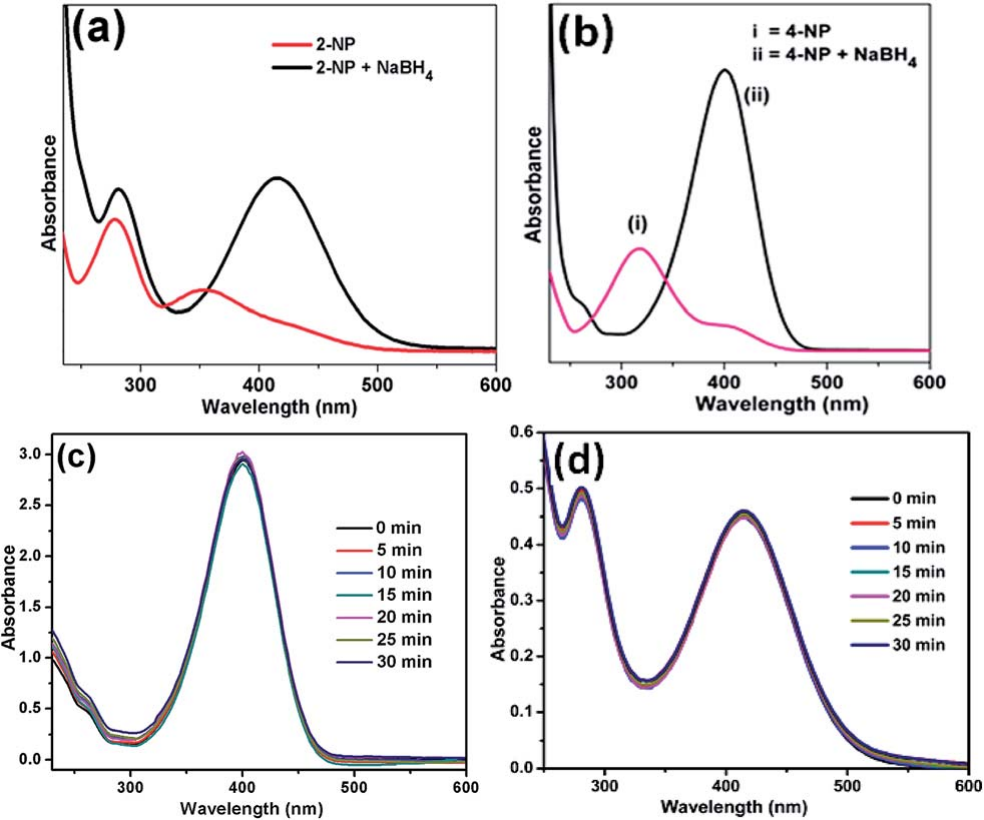
UV-vis spectra of (a) 2-NP and (b) 4-NP before and after adding NaBH_4_ solution and the reduction of (c) 4-NP and (d) 2-NP in the presence of m-CNFs recorded every 5 min.

The catalytic activity of Ag/CNF, Au/CNF and Ni/CNF towards the reduction of 2- and 4-NP was tested. The 2- and 4-NP reduction reaction in the presence of NaBH_4_ is very simple and steady process. However, the reduction process is highly restricted in the absence of metal catalysts due to the high kinetic barrier between the negative ions, nitro group of NP and BH_4_^−^ ions. Initially, blank reactions without any catalysts were carried out; the results are demonstrated in Fig. 6. The pure 2-NP showed a broad band at ∼350 nm. Similarly, the 4-NP demonstrated an intense UV adsorption band at ∼315 nm. Upon the addition of NaBH_4_, the adsorption bands of both 4-NP and 2-NP were noticed to be red-shifted due to the formation of nitrophenolate ions (Fig. 6a and b). In the absence of catalyst, the adsorption band of nitrophenolate ions was unchanged even after 24 h. Similarly, the catalytic activity of the fresh m-CNFs was also tested. However, it was found to be inactive (Fig. 6c and d).

**Fig. 7:**
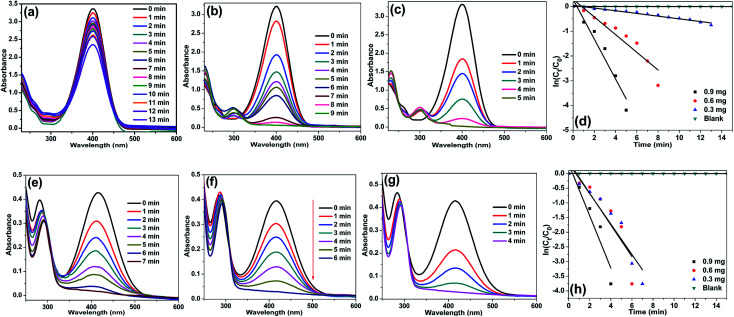
UV-Vis spectra for the reduction of (a–c) 4-NP and (e–g) 2-NP in aqueous solution recorded every 1 min using different amounts of Ni/CNF: (a, e) 0.3 mg, (b, f) 0.6 mg and (c, g) 0.9 mg, and plots of ln[*C*_*t*_/*C*_0_] *versus* reaction time for reduction of (d) 4-NP and (h) 2-NP with NaBH_4_ over Ni/CNFs.

**Fig. 8:**
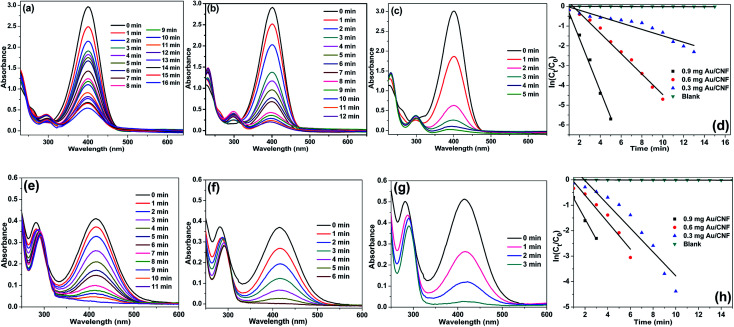
UV-Vis spectra for the reduction of (a–c) 4-NP and (e–g) 2-NP in aqueous solution recorded every 1 min using different amounts of Au/CNF: (a, e) 0.3 mg, (b, f) 0.6 mg and (c, g) 0.9 mg, and plots of ln[*C*_*t*_/*C*_0_] *versus* reaction time for reduction of (d) 4-NP and (h) 2-NP with NaBH_4_ over Au/CNF.

**Fig. 9:**
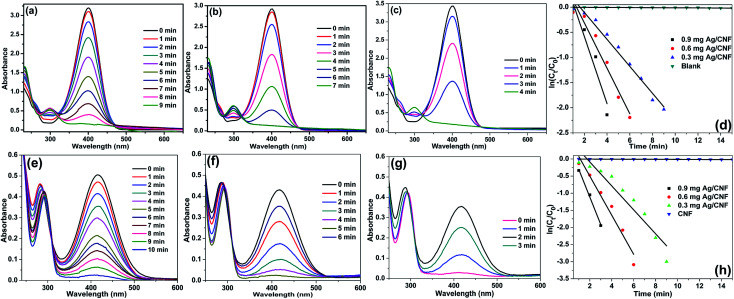
UV-Vis spectra for the reduction of (a–c) 4-NP and (e–g) 2-NP in aqueous solution recorded every 1 min using different amounts of Ag/CNF: (a, e) 0.3 mg, (b, f) 0.6 mg and (c, g) 0.9 mg, and plots of ln[*C*_*t*_/*C*_0_] *versus* reaction time for reduction of (d) 4-NP and (h) 2-NP with NaBH_4_ over Ag/CNF.

To our delight, in the presence of Ag/CNF, Au/CNF and Ni/CNF, the both 4- and 2-NP reduced very rapidly (Fig. 7–9). The reduction products were confirmed by UV-vis ((Fig. 8 and 9) and UHPLC analysis (Fig. S4–S11†)). It was found that even a very low amount of catalyst (0.3, 0.6 and 0.9 mg) is enough for the complete reduction of nitrophenols with high reaction rate. The rate of reaction increased with the amount of catalyst and reaction time. The Ni/CNF showed an excellent activity towards the reduction of both 4- and 2-NP. With 0.3 mg of the Ni/CNF, the system required only 9–10 min for the reduction of both 4- and 2-NP. Increasing the amount of Ni/CNF from 0.3 mg to 0.6 or 0.9 mg showed the rapid reduction of nitrophenols. Especially, with 0.9 mg of catalyst, the Ni/CNF required only 4 to 5 min of the reaction time to achieve the 100% conversion of 4- and 2-NP to 4- and 2-AP. The reaction kinetics was studied using the time-dependent absorption spectra. The reaction rate is assumed to be independent of NaBH_4_ concentration due to its excess use. Alike, the adsorption of 2- and 4-NP on m-CNFs can be also ignored due to its inactiveness. The linear correlation between ln(*C*_*t*_/*C*_0_) and time at 295 K shows that the reduction of 2- and 4-NP by Ni/CNFs follows the pseudo-first-order reaction kinetics (Fig. 3d and h). The kinetic reaction rate constants (*k*_app_) were estimated from the slope of the ln(*C*_*t*_/*C*_0_) *versus* time liner curve. The *k*_app_ values of 2.22, 4.69 and 9.20 × 10^−2^ s^−1^ was calculated for the reduction of 4-NP by Ni/CNF with different weights, 0.3, 0.6 and 0.9 mg, respectively. In case of the reduction of 2-NP by Ni/CNF, the *k*_app_ values were determined to be 6.44 × 10^−2^ s^−1^ (0.3 mg), 10.95 × 10^−2^ s^−1^ (0.6 mg) and 15.68 × 10^−2^ s^−1^ (0.9 mg). For a quantitative comparison, the reaction rate constant per unit mass (*M*_metal_) was calculated by using the equation, the activity parameter *k*′ = *k*/*M*, which is the ratio of the *k*_app_ to the weight of the metal active site added. The high *k*′ value of 719 s^−1^ g^−1^ (4-NP) and 422 s^−1^ g^−1^ (2-NP) was determined for the reduction of NP using 0.9 mg of Ni/CNF. To the best of our knowledge, this is the most efficient catalyst for the reduction of 2- and 4-NP reported to date. The small Ni NPs size, homogenous distribution of Ni NPs on m-CNFs surface and strong metal-support interaction, are the main key factors for the superior activity of Ni/CNF. The present *k*_app_ values are compared over previously reported heterogeneous catalysts (Table 1). According to the previous reports, the unsupported Ni NPs require at least 0.5 h to 3 h of reaction time for the reduction of 4- and 2-NP under present reaction conditions.^27^ Zhu *et al.*^28^ prepared of Ni NPs in spherical polyelectrolyte brush nanoreactor (Ni–NPs/SPB) and their catalytic activity towards the reduction of 4-NP. They found that the Ni–NPs/SPB is highly efficient and the rate constant (*k*_app_) value of 1.56 × 10^−4^ s^−1^ was calculated. Similarly, Jiang and co-workers^29^ prepared nano-Ni core mesoporous–silica shell particles (Ni@SiO_2_) for the reduction of 4-NP. The Ni@SiO_2_ system required at least 16 min for the complete reduction of 4-NP. The maximum *k*_app_ value was calculated to be 2.818 × 10^−3^ s^−1^. Moreover, nickel nanoparticles supported reduced graphene oxide (Ni–RGO) catalyst^30^ was tested for the reduction of 4-NP and 2-NP under the present reaction conditions (Fig. S12†). In comparison to the present Ni/CNF, the Ni–RGO system required longer reaction time of 9 min for the complete conversion of 4-NP to 4-AP (with *k*_app_ value of 5.57 × 10^−3^ s^−1^). Similarly, the Ni–RGO system required 7 min for the reduction of 2-NP to 2-AP and the *k*_app_ value was calculated to be 6.33 × 10^−3^ s^−1^.

**Table 1:** Comparison of present cellulose nanocomposites over other heterogeneous catalysts^a^

S. no.	Catalyst	Amount of catalyst	Reaction time	Compound	*k*_app_	Reference
1	Ni–NPs composite brushes	0.163 mg L^−1^	60 min	4-NP	1 × 10^−3^ s^−1^	28
2	Ni NPs	90 mg	16 min	4-NP	2.7 × 10^−3^ s^−1^	27
**3**	**Ni/CNF**	**0.9 mg**	**4 min**	**2-NP**	**15.68 × 10** ^**−2**^ **s** ^**−1**^	**[#]**
**4**	**Ni/CNF**	**0.9 mg**	**5 min**	**4-NP**	**9.20 × 10** ^**−2**^ **s** ^**−1**^	**[#]**
5	Fe@Au–ATPGO	1.8 g L^−1^	25 min	4-NP	0.84 × 10^−5^ (mol L^−1^ min^−1^)	37
6	Fe@Au–ATPGO	1.8 g L^−1^	25 min	2-NP	0.74 × 10^−5^ (mol L^−1^ min^−1^)	37
7	Ni/MC-750	0.7 mg	10 min	4-NP	6.26 × 10^−3^ s^−1^	38
8	AuNPs/SNTs	8 mg	5 min	4-NP	10.64 × 10^−3^ s^−1^	31
9	Au/PMMA	3.5 mg	10 min	4-NP	7.2–7.9 × 10^−3^ s^−1^	32
10	Au@SiO_2_	—	60 min	4-NP	4.6 × 10^−4^ s^−1^	39
**11**	**Au/CNF**	**0.9 mg**	**5 min**	**4-NP**	**9.05 × 10** ^**−2**^ **s** ^**−1**^	**[#]**
**12**	**Au/CNF**	**0.9 mg**	**3 min**	**2-NP**	**11.34 × 10** ^**−2**^ **s** ^**−1**^	**[#]**
13	Fe_3_O_4_@SiO_2_–Ag MNPs	3 mg	14 min	4-NP	0.83 × min^−1^	33
14	Ag/carbon fiber	1.0 mg	8 min	4-NP	4.2 × 10^−3^ s^−1^	36
15	Fe_3_O_4_/SiO_2_@Ag	0.02 mg	2 min	4-NP	2.5 × 10^−2^ s^−1^	40
16	Fe_3_O_4_/SiO_2_@Ag	0.02 mg	5 min	2-NP	5.5 × 10^−3^ s^−1^	40
17	Ag10@SBA-15	0.9 mg	6 min	4-NP	1.274 × 10^−2^ s^−1^	41
**18**	**Ag/CNF**	**0.9 mg**	**4 min**	**4-NP**	**9.05 × 10** ^**−2**^ **s** ^**−1**^	**[#]**
**19**	**Ag/CNF**	**0.9 mg**	**3 min**	**2-NP**	**11.34 × 10** ^**−2**^ **s** ^**−1**^	**[#]**
20	Ni NPs–(p(AMPS)) hydrogel	48 mg	60 min	4-NP	5.63 × 10^−2^ s^−1^	42
21	Ni NPs–(p(AMPS)) hydrogel	48 mg	60 min	2-NP	3.07 × 10^−2^ s^−1^	42
22	Ni@Au/nano-silica	2 mg	9.6 min	4-NP	8.3 × 10^−3^ s^−1^	43
23	Ni NPs/silica	7 mg	16 min	4-NP	2.818 × 10^−3^ s^−1^	44
24	Au/graphene	0.1 mg	12 min	4-NP	3.17 × 10^−3^ s^−1^	45
25	Au NPs	6 mg	13 min	4-NP	2.1 × 10^−3^ s^−1^	46
26	Au/MWCNTs	1 mg	120 min	4-NP	1.1 × 10^−4^ s^−1^	47
27	Ag(seed)–SiO_2_(*p*-TSA^−^)	250 mg L^−1^	26 min	4-NP	2.48 × 10^−3^ s^−1^	48
28	Ag–NP/C	1.0 mg	25 min	4-NP	1.69 × 10^−3^ s^−1^	49
29	Ag/alumina	0.3 mg	14 min	4-NP	3.2 × 10^−3^ s^−1^	50
30	Ag@AMH	4 beads	13 min	4-NP	0.27 min^−1^	51
31	NiO/CNP	2 mg	2 min	4-NP	4.2 × 10^−2^ s^−1^	52
32	Ag/HHP	1 mg	9 min	4-NP	5.94 × 10^−2^ s^−1^	53
33	Ni/HHP	2.5 mg	22 min	2-NP	6.19 × 10^−4^ s^−1^	54

a [#] = present work.

Fig. 8 shows the UV-Vis spectra for the reduction of 4-NP and 2-NP in aqueous solution recorded every 1 min using different amounts of Au/CNF. The results confirmed that the Au/CNF is highly effective for the reduction of 4- and 2-NP. In the reduction of 4-NP, 0.3 mg of Au/CNF was found to be not enough since the catalytic system could not achieve the complete reduction of 4-NP even after 30 min. However, the complete reduction of 4-NP was attained with 0.6 mg or 0.9 mg of the Au/CNF. The conversion of 2-NP to 2-AP was also highly effective with low amount of catalyst. The *k*_app_ values of 2.15, 4.98 and 9.78 × 10^−2^ s^−1^ was determined for the reduction of 4-NP by Au/CNF with different weights, 0.3, 0.6 and 0.9 mg, respectively. In case of the reduction of 2-NP by Au/CNF, the *k*_app_ values were calculated to be 6.46 × 10^−2^ s^−1^ (0.3 mg), 11.56 × 10^−2^ s^−1^ (0.6 mg) and 12.56 × 10^−2^ s^−1^ (0.9 mg). In addition, the reaction rate constant per unit mass (*M*_Au_) was calculated to be 957 and 1200 s^−1^ g^−1^ for the Au/CNF-mediated reduction of 4-NP and 2-NP, respectively. The values (957 and 1200 s^−1^ g^−1^) were found to be better when compared to silica supported Ni@Au bimetallic catalyst (307 and 237 s^−1^ g^−1^) (Table 1). Alike, Zhang *et al.*^31^ prepared Au NPs supported electrospun silica nanotubes (AuNPs/SNTs nanocomposite) for the reduction of *p*-nitrophenol. The AuNPs/SNTs nanocomposite reduced the 4-NP within 280 s with the kinetic constants *k*_app_ of 10.64 × 10^−3^ s^−1^, which was much higher than other substrate supported Au nanocatalysts. However, the present cellulose nanocomposites (Ni/CNF, the Au/CNF and Ag/CNF) showed better catalytic activity than the AuNPs/SNTs nanocomposite. Gold nanoparticles (Au NPs) deposited poly(methyl methacrylate) (PMMA) composites showed that the rate constant and the activation energy are estimated to be 7.2–7.9 × 10^−3^ s^−1^ at 295 K and 38 kJ mol^−1^, respectively.^32^ Magnetically recoverable Au nanocatalyst was employed for the reduction of 4-NP by Chang. The Au nanocatalyst demonstrated excellent rate constant *k*_app_ of 31–78 × 10^−2^ m^−1^.^32^ The Au NPs anchored carbon nanotube (Au/MWCNTs)^47^ catalyst was tested under present reaction conditions (Fig. S13†). However, the Au/MWNCTs did not achieve the complete conversion of 4-NP even after 30 min of the reaction time. The result confirmed that the cellulose nanofibers are much suitable support for the decoration Au NPs when compared to MWCNTs.

Alike the Ni/CNF and Au/CNF, Ag/CNF also found to highly active toward the reduction of 4- and 2-NP. Fig. 9 shows the adsorption spectra for the reduction of 4- and 2-NP in aqueous solution recorded every 1 min using different amounts of Ag/CNF. So far, Ag-based nanocatalysts are one of the best catalysts for the reduction of nitrophenols. For example, Ag NPs supported on halloysite nanotubes was prepared and used for the reduction of 4-NP by Liu and co-workers.^34^ Similarly, Ag-deposited silica-coated Fe_3_O_4_ magnetic nanoparticle (Fe_3_O_4_@SiO_2_–Ag) for the reduction of *p*-nitrophenol was reported by Du *et al.*^35^ For the complete reduction of 4-NP, the Fe_3_O_4_@SiO_2_–Ag took 14 min of the reaction time and the *k*_app_ value was determined to be 0.83–0.68 min^−1^. Recently, Zhang *et al.*^36^ prepared AgNPs/electrospun carbon nanofibers (CNFs/AgNPs) for the reduction of 4-NP. The minimum time required for the reduction of 4-NP was 8 min and the *k*_app_ value was calculated to be 6.2 × 10^−3^ s^−1^ at 295 K. To our delight, the present cellulose nanofiber supported Ag NPs catalyst (Ag/CNF) showed better results than the previous reports. In the reduction of 4-NP, 0.6 mg of Au/CNF was found to be enough since the catalytic system achieved the complete reduction of 4-NP after only 12–6 min. When increasing the amount of Au/CNF from 0.6 to 0.9, the 4-NP reduced with high rate constant. The time required for the reduction of 4-NP was just 5 min. Similarly, the conversion of 2-NP to 2-AP was also highly effective with low amount of catalyst (Fig. 9e–h). The *k*_app_ values of 2.76, 4.35 and 9.05 × 10^−2^ s^−1^ was determined for the reduction of 4-NP by Au/CNF with different weights, 0.3, 0.6 and 0.9 mg, respectively. In case of the reduction of 2-NP by Au/CNF, the *k*_app_ values were calculated to be 5.67 × 10^−2^ s^−1^ (0.3 mg), 10.71 × 10^−2^ s^−1^ (0.6 mg) and 11.34 × 10^−2^ s^−1^ (0.9 mg). In addition, the reaction rate constant per unit mass (*M*_Ag_) was calculated to be 395 and 501 s^−1^ g^−1^ for the Ag/CNF-catalyzed reduction of 4-NP and 2-NP, respectively. In addition, the Ag/alumina catalyst^49^ was tested for the reduction of 4-NP and 2-NP under the optimized reaction conditions (Fig. S14†). The Ag/alumina system required 10 min for the complete conversion of 4-NP to 4-AP (with *k*_app_ value of 5.57 × 10^−3^ s^−1^), whereas the present Ag/CNF achieved it in 5 min. In case of the complete reduction of 2-NP to 2-AP, the Ag/alumina required 8 min whereas the present Ag/CNF required just 3 min.

Overall, the present cellulose nanocomposites are found to be highly effective when compared to previously reported systems (refer Table 1). We conclude that the cellulose nanofiber could have provided a better interaction with meal NPs. The other possible reasons for the better catalytic activity are; (1) small size of the metal nanoparticles, (2) strong interaction between metal nanoparticles and support, (3) nature of support and (4) high surface area. To the best of our knowledge, the present cellulose nanocomposites are the most efficient catalysts for the reduction of 4- and 2-NP reported to date (Table 1).

The possible mechanism for the reduction of nitrophenols has been previously reported.^40,41^ The complete reduction process occurs mainly on the surface of metal NPs supported on the surface of cellulose nanofibers. In the reduction of nitrophenols, BH_4_^−^ act as donor and the nitro group act as acceptor. In the first step, the nitrophenolate ion adsorbs on the surface of metal catalyst and forms active hydrogen atoms. In the reduction process, the catalytic NPs could acts as an electronic relay system and speed up the electron transfer from BH_4_^−^ to nitro groups and the catalytic system subsequently gives aminophenols.

## Conclusions

In conclusion, highly active Au, Ag and Ni NPs supported cellulose nanofiber composites (Au/CNF, Ni/CNF and Ag/CNF) were successfully prepared. The resultant nanocomposites demonstrated an excellent catalytic activity towards the reduction of 4- and 2-NP water. Even a very low amount of catalyst (0.6–0.9 mg) was also found to be good enough to achieve 100% reduction of 4- and 2-NP with higher reaction rate (within 5 min). The present catalyst showed high rate constant (*k*_app_) and good reaction rate constant per unit mass (*k*′) values. To the best our knowledge, Au/CNF, Ni/CNF and Ag/CNF are the most efficient nanocatalysts for the reduction of 4- and 2-NP reported to date.

## Conflicts of interest

There are no conflicts of interest to declare.

## Supplementary Material

RA-008-C7RA10489H-s001
