# Supplementary material for: Gold, silver and nickel nanoparticle anchored cellulose nanofiber composites as highly active catalysts for the rapid and selective reduction of nitrophenols in water
Source: RSC Adv. 2018 Jan 17;8(6):3014–23. doi: 10.1039/c7ra10489h (PMC9077553; doi:10.1039/c7ra10489h)
Supplement: RA-008-C7RA10489H-s001 [file RA-008-C7RA10489H-s001.pdf]

Electronic Supplementary Material (ESI) for RSC Advances.
This journal is © The Royal Society of Chemistry 2018

Supporting Information

Gold, silver and nickel nanoparticles anchored cellulose nanofiber composites as highly active catalysts for the rapid and selective reduction of nitrophenols in water

Mayakrishnan Gopiraman,[a] Dian Deng,[b] Somasundaram Saravanamoorthy,[c] Ill-Min Chung,[a,*] and Ick Soo Kim,[b,*]

[a]*Department of Applied Bioscience, College of Life & Environment Science, Konkuk University, 120 Neungdong-ro, Gwangjin-gu, Seoul 05029, South Korea. E-mail: imcim@konkuk.ac.kr, illminchung@gmail.com*

[b]*Nano Fusion Technology Research Group, Division of Frontier Fibers, Institute for Fiber Engineering (IFES), Interdisciplinary Cluster for Cutting Edge Research (ICCER), Shinshu University, Tokida 3-15-1, Ueda, Nagano prefecture, 386-8567, Japan. E-mail: kimicksoo.gr@gmail.com*

[c]*Department of Chemistry, National Institute of Technology, Tiruchirappalli 620 015, India*

Table S1 Metal content of the nanofiber composites determined by SEM-EDS and ICP-MS analysis

	Catalysts		
	**Au/CNF**	**Ni/CNF**	**Ag/CNF**
	ICP-MS data (metal content)		
	9.45 wt%	21.45 wt%	22.94 wt%
	SEM-EDS data (metal content)		
Spot 1	10.9 wt%	21.8 wt%	22.9 wt%
Spot 2	9.5 wt%	20.8 wt%	21.5 wt%
Spot 3	8.7 wt%	21.9 wt%	23.4 wt%
Average	9.7 wt%	21.8 wt%	22.6 wt%

Table S2. UHPLC quantitative analysis of aminophenol and nitrophenol before and after reduction reaction (See **Fig S4-S11** and **Fig S15**)

S. No	Catalyst	4-NP		4-AP	
		**before**	**after**	**before**	**after**
1	Ni/CNF	0.114 mg	0.0	0.0	0.0869 mg
2	Au/CNF	0.113 mg	0.0	0.0	0.0867 mg
3	Ag/CNF	0.121 mg	0.0	0.0	0.0919 mg
		**2-NP**		**2-AP**	
4	Ni/CNF	0.050 mg	0.0	0.0	0.041 mg
5	Au/CNF	0.062 mg	0.0	0.0	0.049 mg
6	Ag/CNF	0.048 mg	0.0	0.0	0.039 mg

Calculated using UHPLC peak area

Table S3. UV-vis quantitative analysis of aminophenol and nitrophenol before and after reduction reaction (See **Fig S16-20**)

S. No	Catalyst	4-NP		4-AP	
		**before**	**after**	**before**	**after**
1	Ni/CNF	0.113 mg	0.0	0.0	0.0865 mg
2	Au/CNF	0.107 mg	0.0	0.0	0.0817 mg
3	Ag/CNF	0.123 mg	0.0	0.0	0.0922 mg
		**2-NP**		**2-AP**	
4	Ni/CNF	0.047 mg	0.0	0.0	0.043 mg
5	Au/CNF	0.057 mg	0.0	0.0	0.049 mg
6	Ag/CNF	0.046 mg	0.0	0.0	0.039 mg

Calculated using UV absorbance

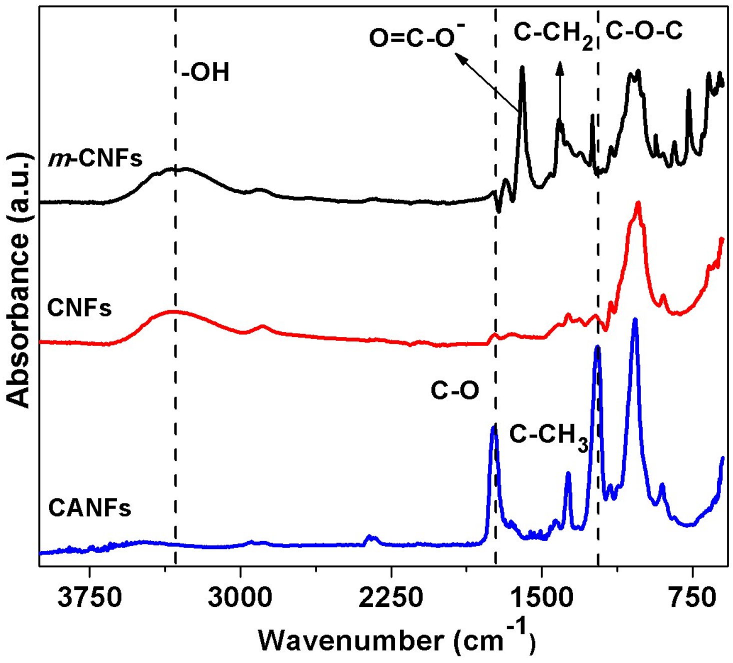

Fig. S1. FTIR spectra of CANFs, CNFs and *m*-CNFs (Reprinted with permission from ref. 1. Copyright 2015, Elsevier.)

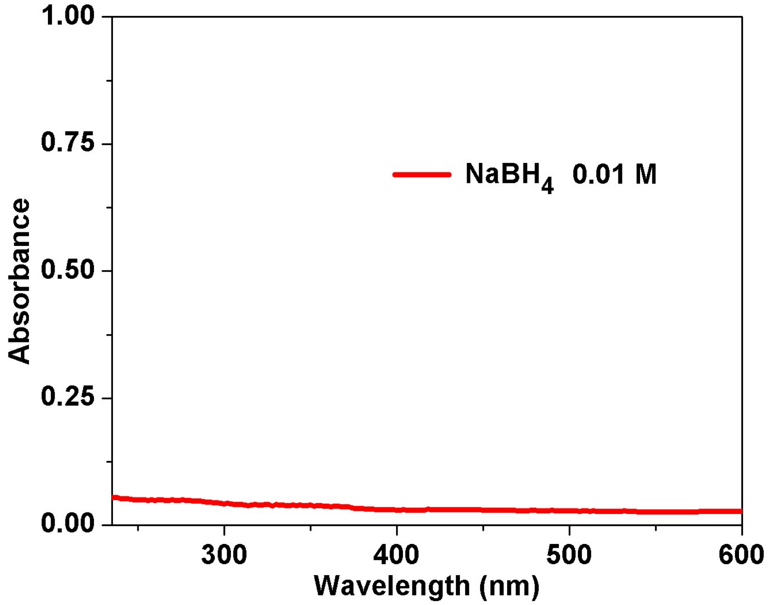

Fig. S2. UV-vis spectrum of $NaBH_4$ in aqueous solution (0.01 M).

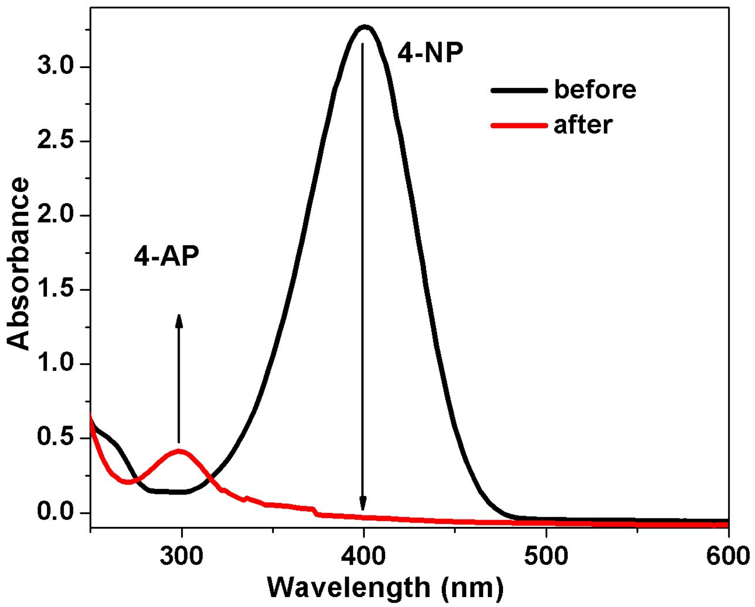

Fig. S3. UV–Vis spectra for the reduction of 4-NP before and after reduction reaction.

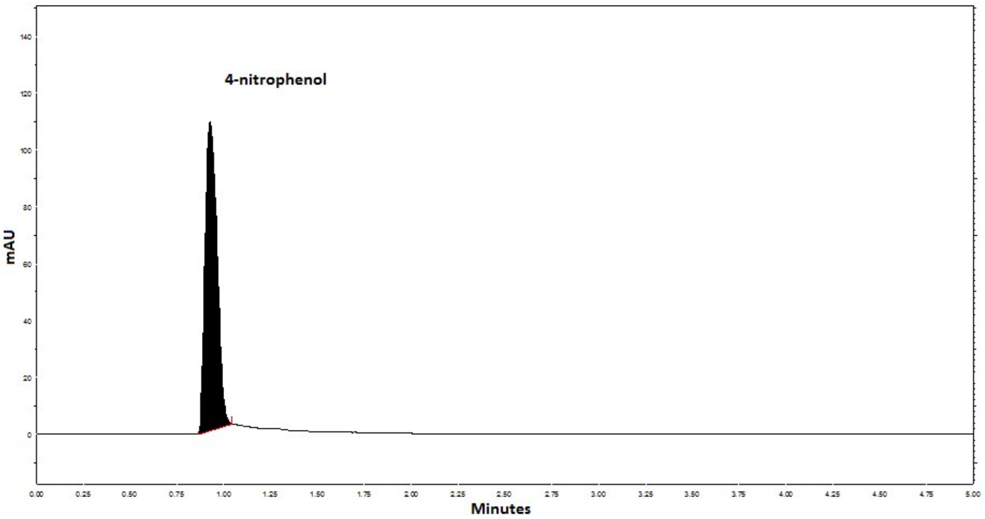

Fig. S4. UHPLC chromatogram of 4-nitrophenol in aqueous solution.

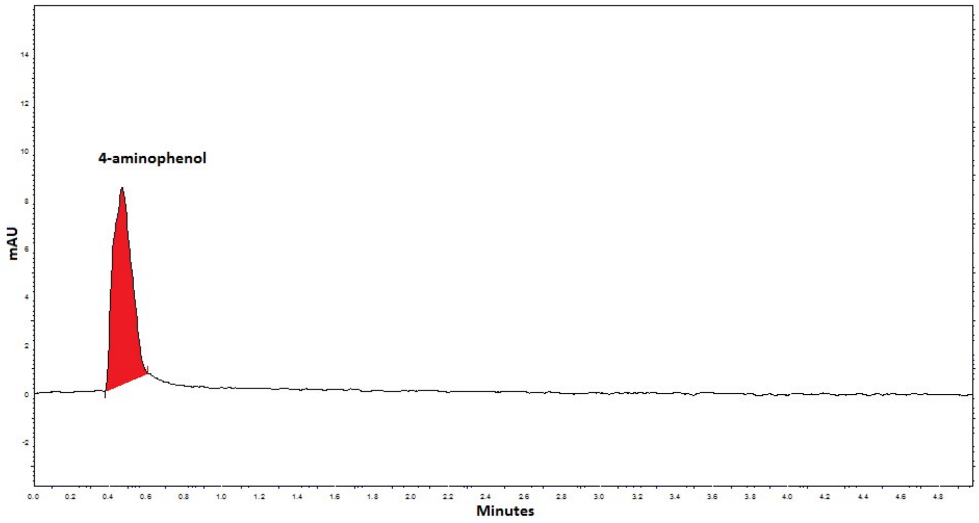

Fig. S5. UHPLC chromatogram of reductive product of 4-nitrophenol catalyzed by Ni/CNF.

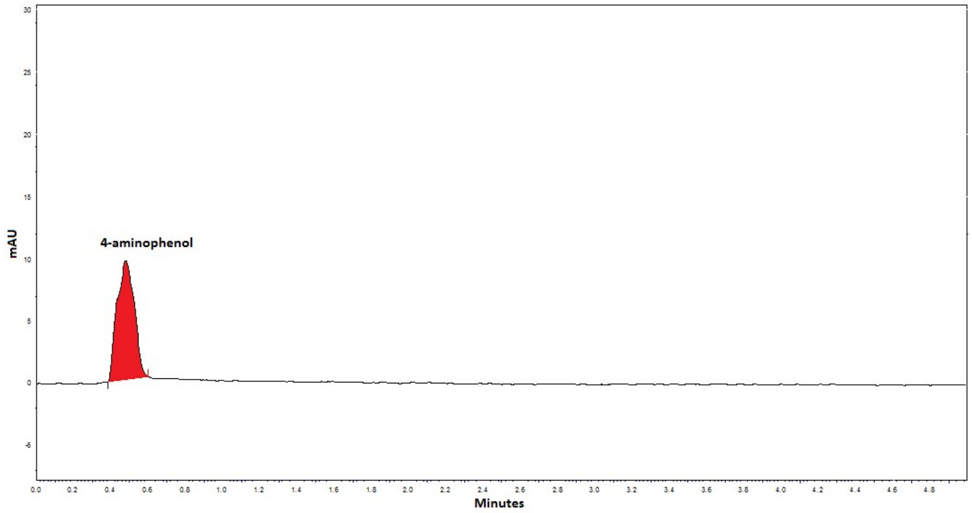

Fig. S6. UHPLC chromatogram of reductive product of 4-nitrophenol catalyzed by Au/CNF.

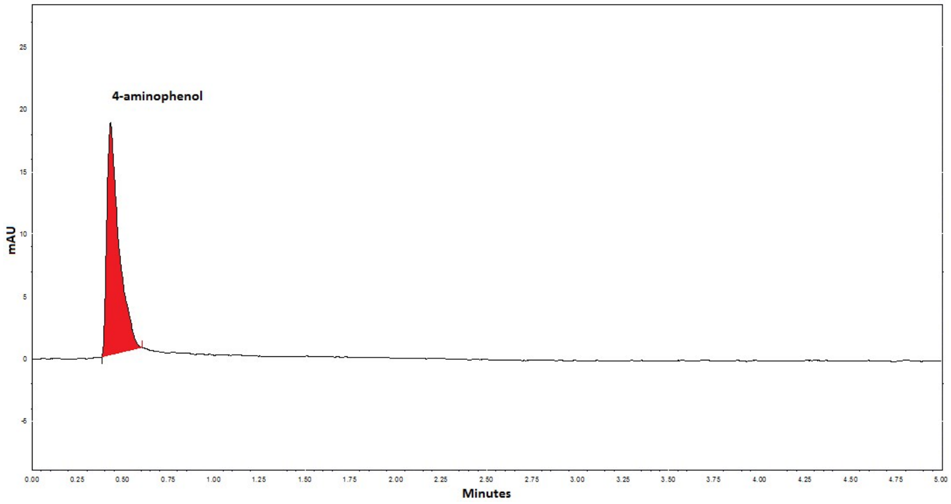

Fig. S7. UHPLC chromatogram of reductive product of 4-nitrophenol catalyzed by Ag/CNF.

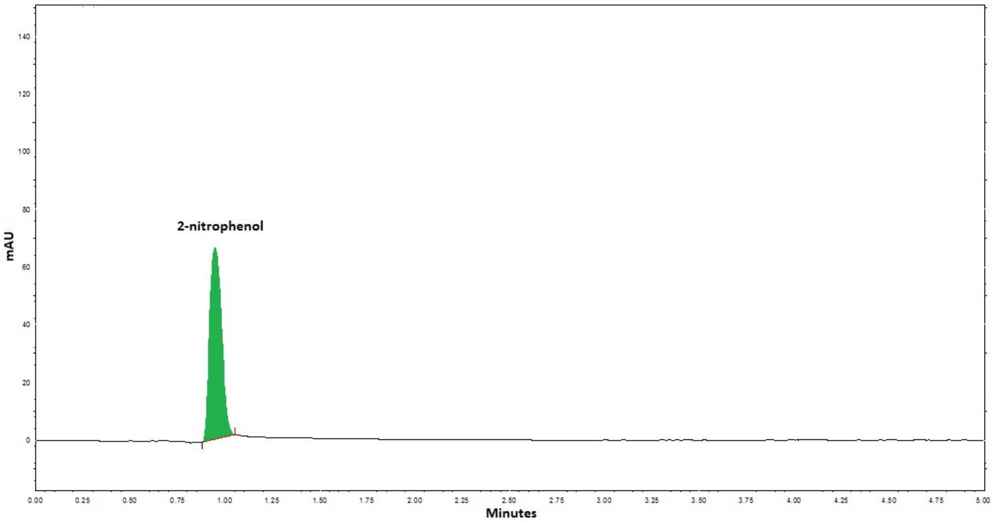

Fig. S8. UHPLC chromatogram of 2-nitrophenol in water.

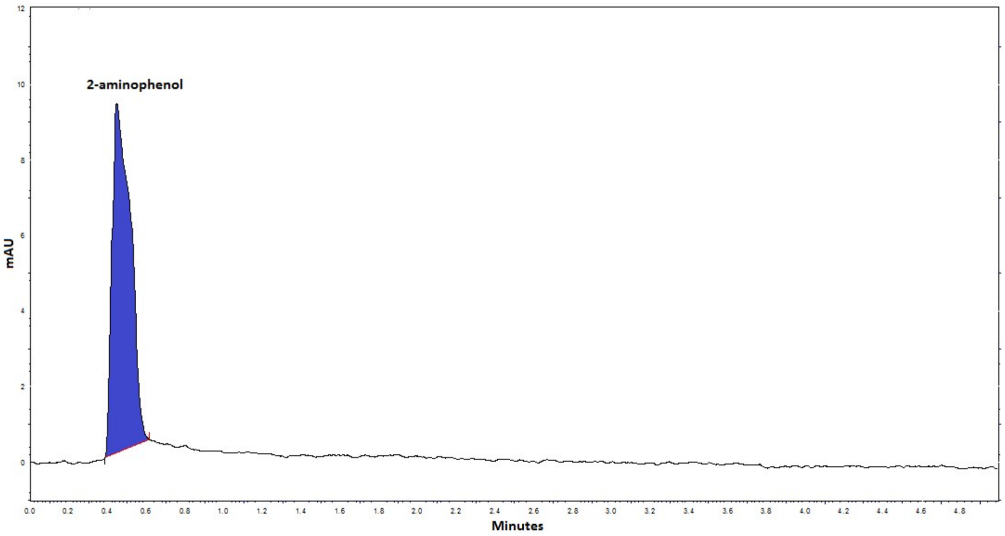

Fig. S9. UHPLC chromatogram of reductive product of 2-nitrophenol catalyzed by Ni/CNF.

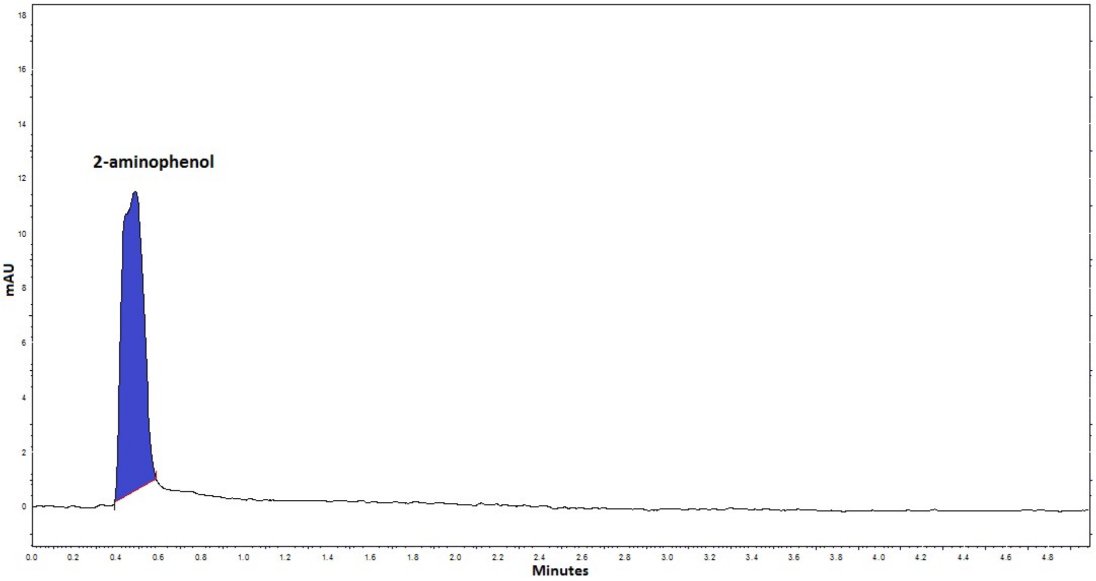

Fig. S10. UHPLC chromatogram of reductive product of 2-nitrophenol catalyzed by Au/CNF.

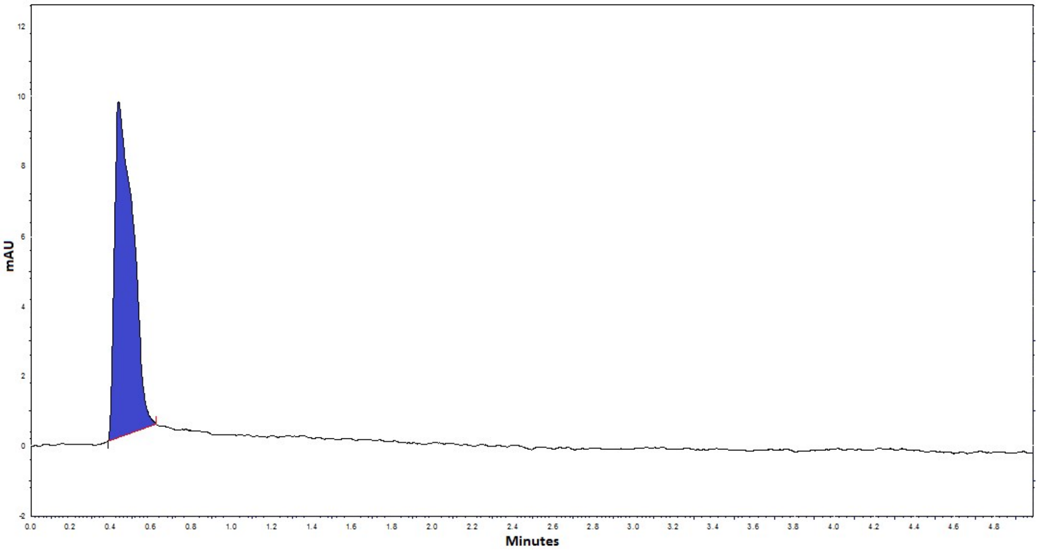

Fig. S11. UHPLC chromatogram of reductive product of 2-nitrophenol catalyzed by Ag/CNF.

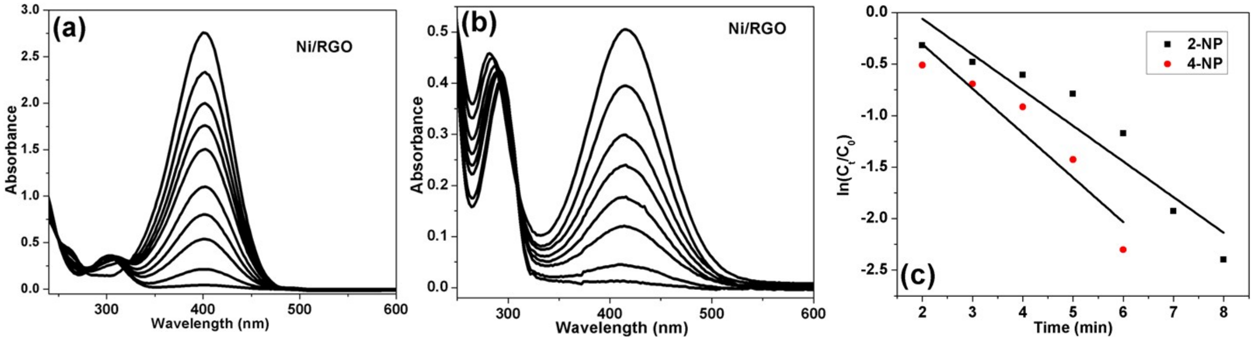

Fig. S12. UV–Vis spectra for the reduction of (a) 4-NP and (b) 2-NP in aqueous solution recorded every 1 min using 0.9 mg of Ni/RGO, and (c) plots of ln[C_t/C_0] *versus* reaction time for reduction of 4-NP and 2-NP with $NaBH_4$ over Ni/RGO.

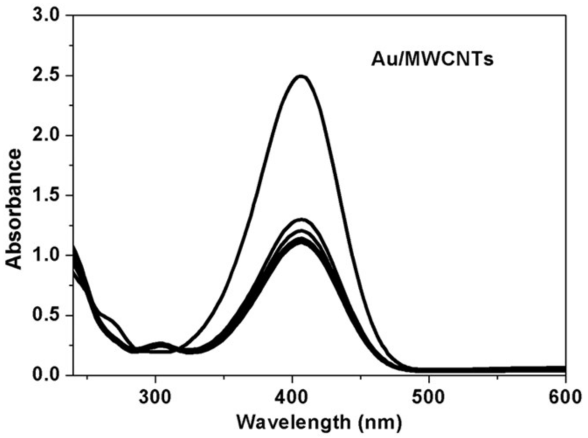

Fig. S13. UV–Vis spectra for the reduction of 4-NP in aqueous solution recorded every 5 min using 0.9 mg of Au/MWCNTs.

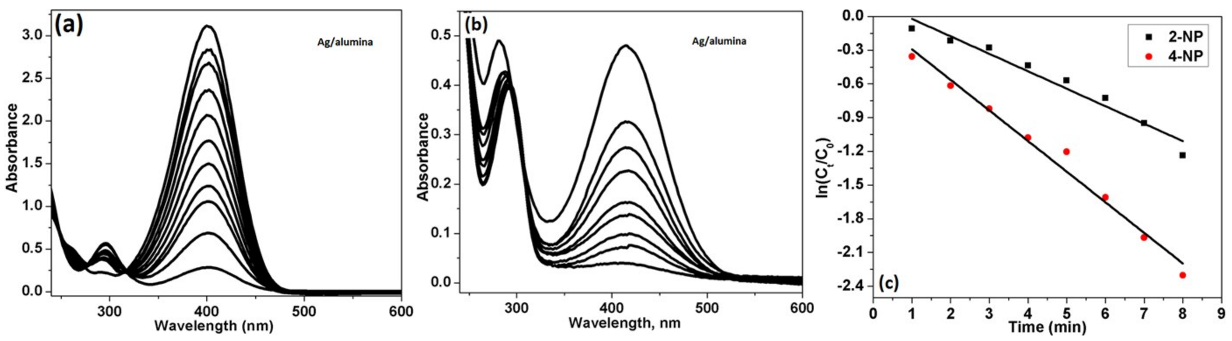

Fig. S14. UV–Vis spectra for the reduction of (a) 4-NP and (b) 2-NP in aqueous solution recorded every 1 min using 0.9 mg of Ag/alumina, and (c) plots of ln[C_t/C_0] *versus* reaction time for reduction of 4-NP and 2-NP with $NaBH_4$ over Ag/alumina.

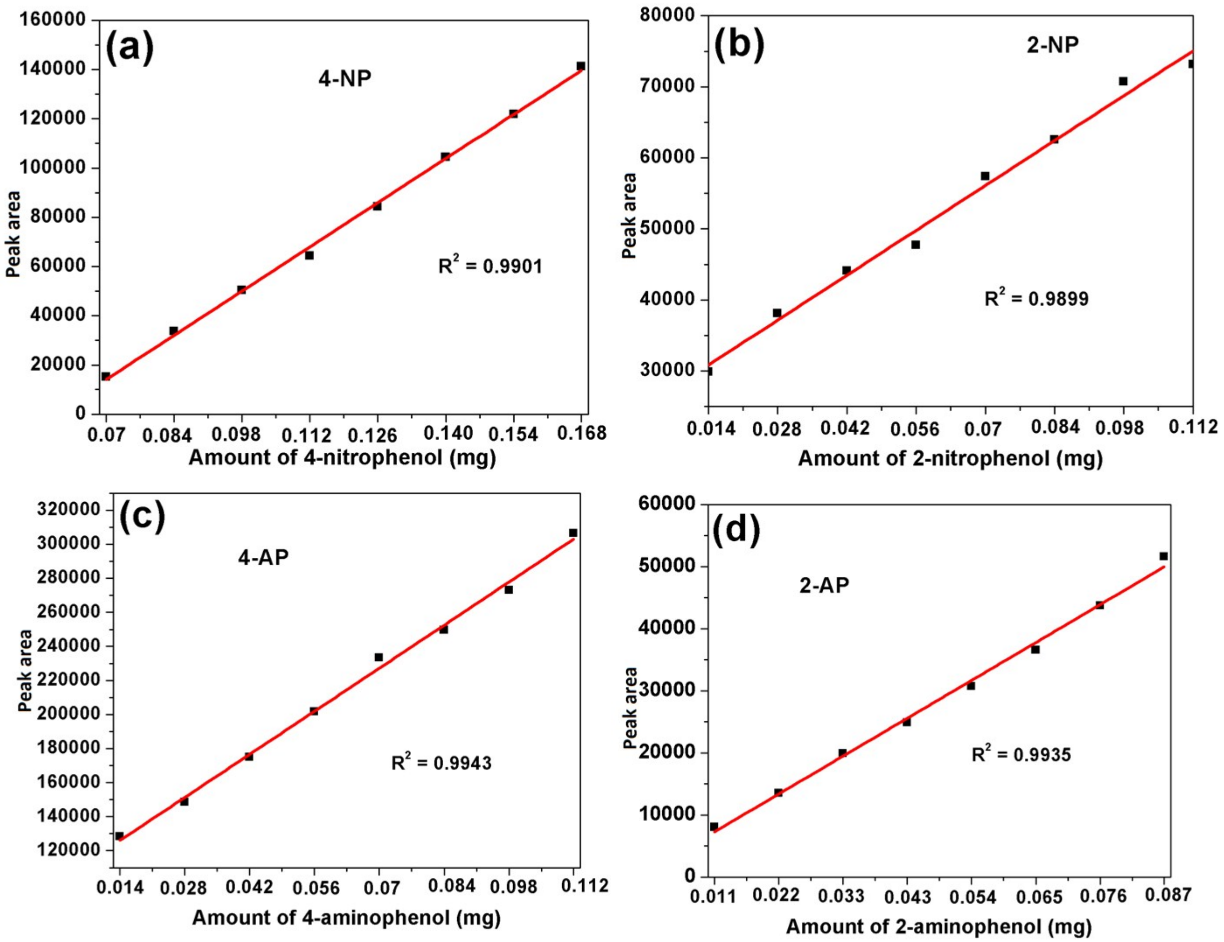

Fig S15. Plots of UHPLC peak area *versus* amount of (a) 4-nitrophenol, (b) 2-nitrophenol, (3) 4-aminophenol and (d) 2-aminophenol.

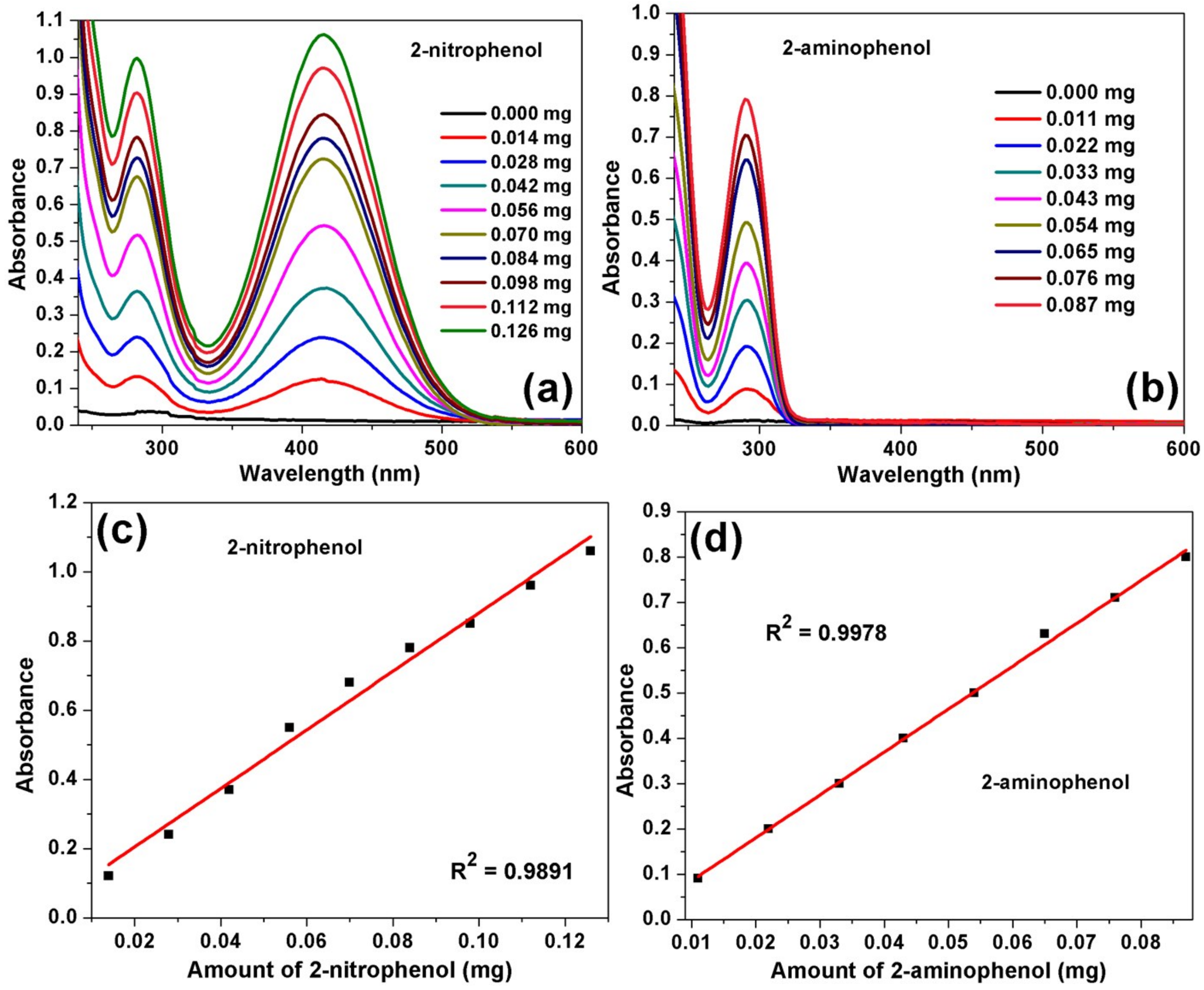

Fig S16. UV–Vis spectra of (a) 2-nitrophenol and (b) 2-aminophenol in aqueous $NaBH_4$ solution, and plots of UV absorbance *versus* amount of (c) 2-nitrophenol and (d) 2-aminophenol.

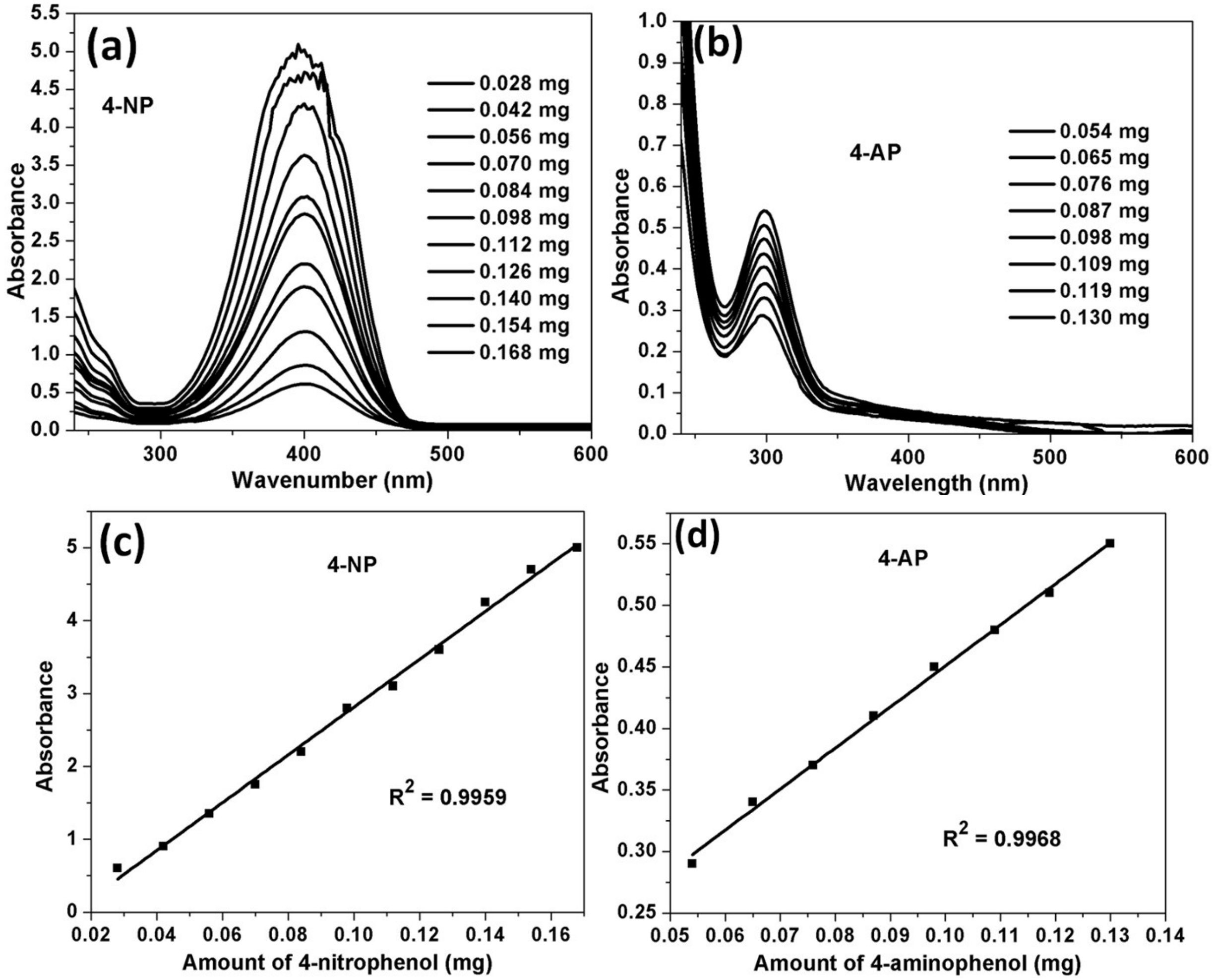

Fig S17. UV–Vis spectra of (a) 4-nitrophenol and (b) 4-aminophenol in aqueous $NaBH_4$ solution, and plots of UV absorbance *versus* amount of (c) 4-nitrophenol and (d) 4-aminophenol.

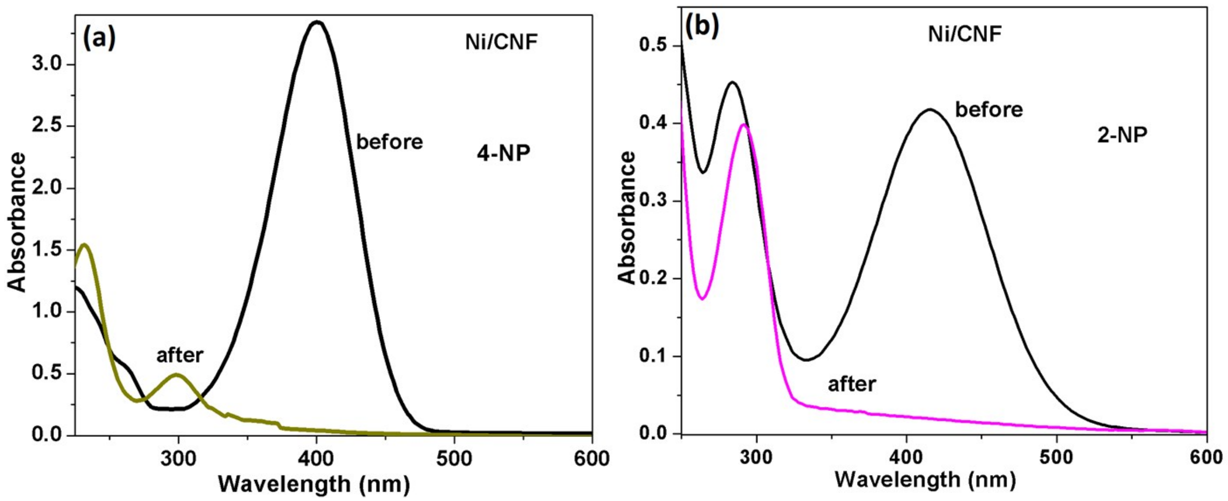

Fig. S18. UV–Vis spectra for the reduction of (a) 4-NP and (b) 2-NP in aqueous solution recorded before and after addition 0.9 mg of Ni/CNF.

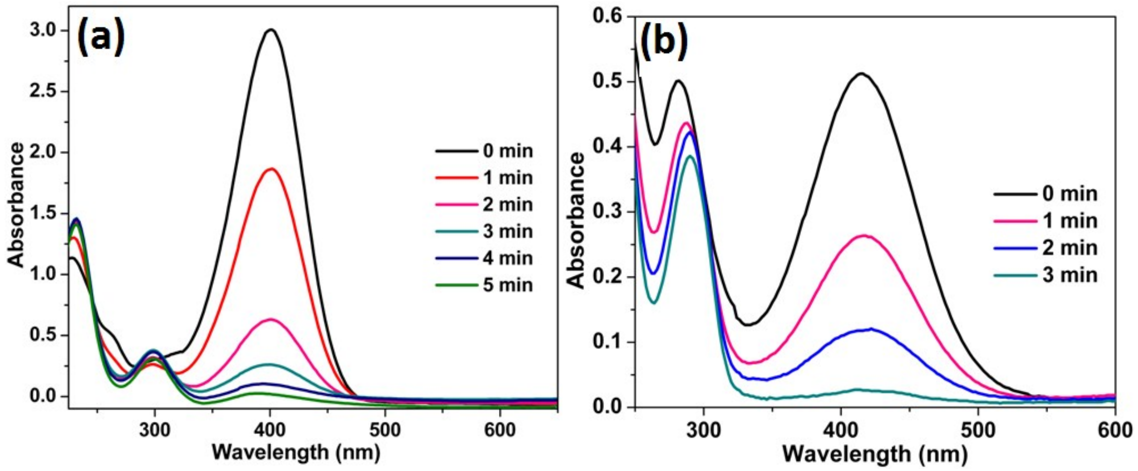

Fig. S19. UV–Vis spectra for the reduction of (a) 4-NP and (b) 2-NP in aqueous solution recorded every 1 min using 0.9 mg of Au/CNF.

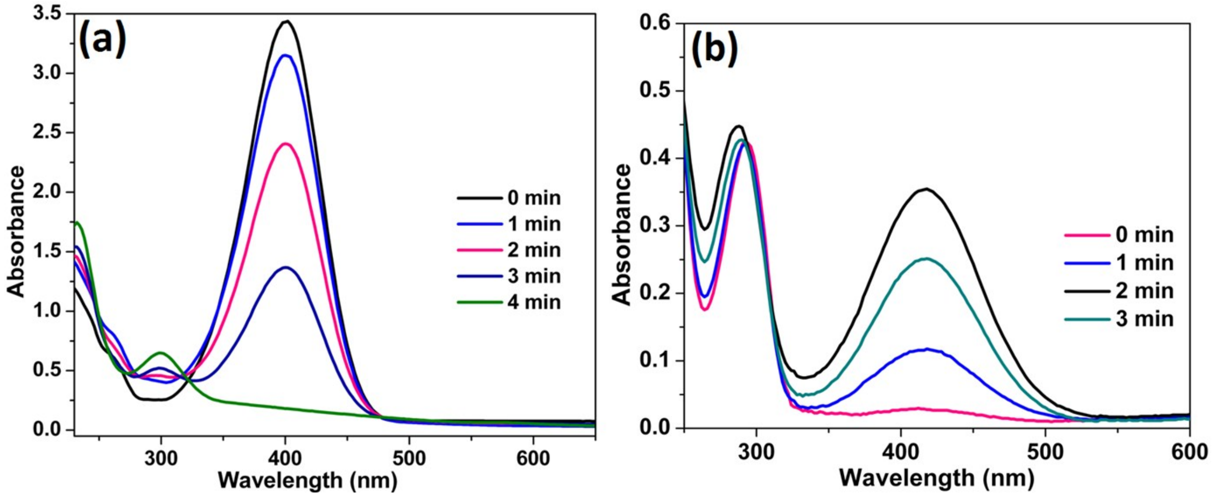

Fig. S20. UV–Vis spectra for the reduction of (a) 4-NP and (b) 2-NP in aqueous solution recorded every 1 min using 0.9 mg of Ag/CNF.

References:

1. M. Gopiraman, H. Bang, G. Yuan, C. Yin, K. H. Song, J. S. Lee, I. M. Chung, R. Karvembu and I. S. Kim, *Carbohydr Polym.*, 2015, **132**, 554-564.
